# To be or not to be a synonym – revision of the *Donaciaclavareaui*-*fukiensis* complex (Coleoptera, Chrysomelidae, Donaciinae)

**DOI:** 10.3897/zookeys.856.32388

**Published:** 2019-06-17

**Authors:** Elisabeth Geiser

**Affiliations:** 1 St.-Julien-Strasse 2/314, 5020 Salzburg, Austria Unaffiliated Salzburg Austria

**Keywords:** China, Fujian, East Palaearctic, *
Donacia clavareaui
*, *
Donacia fukiensis
*, *
Donacia kweilina
*, *
Donacia mediohirsuta
*, identification key, lectotype, Museum Frey, reed beetles, synonym, taxonomy

## Abstract

The East Palaearctic species *Donaciaclavareaui* Jacobson, 1906 and *Donaciafukiensis* Goecke, 1944 have been confused for decades. Finally, *D.fukiensis* was synonymized with *D.clavareaui* by Askevold (1990) but he could not examine the type series of *D.fukiensis* because it was stored in an inaccessible collection. Cong and Yu (1997) re-established *D.fukiensis* as a distinct species, also without direct access to the type series. The synonymization by Askevold (1990) was applied in the identification key of Palaearctic Chrysomelidae (Warchalowski 2010) and the Catalogue of Palaearctic Chrysomelidae (Silfverberg 2010). Because the type series of *D.fukiensis* is now accessible, it has been possible to proof that *D.fukiensis* is a distinct species, and a lectotype has been established from the series of seven syntypes. *Donaciakweilina* Chen, 1966 and *D.mediohirsuta* Chen, 1966, which were split from the mixture of *D.clavareaui* and *D.fukiensis*, are now also synonymized with *D.clavareaui*, because their characters are the same or within the variation range of the characters of *D.clavareaui.* Furthermore, a distribution map is provided with the reliable records known to date.

## Introduction

The East Palaearctic species of *Donaciaclavareaui* Jacobson, 1906, *D.fukiensis* Goecke, 1944, *D.kweilina* Chen, 1966, and *D.mediohirsuta* Chen, 1966 all have in common that their pronotum is pubescent while their elytra are glabrous. All other East Palaearctic *Donacia* species have either hairs on both pronotum and elytra or no hairs.

Although the first descriptions of *D.clavareaui* and of *D.fukiensis* are very detailed (see Appendix 1, 2) it is not possible to distinguish these two species with the described characters alone. Worse, each description leads to *D.clavareaui* and to *D.fukiensis* without any contradiction. Therefore many misidentifications occurred, especially in specimens from China. Subsequently in the identification key of Gressitt and Kimoto (1961) only *D.fukiensis* was considered to occur in China, which resulted in further identification errors. Chen (1966) split *D.kweilina* and *D.mediohirsuta* from this mixture. Askevold (1990) synonymized *D.fukiensis* with *D.clavareaui*. Cong and Yu (1997) re-established *D.fukiensis* as a distinct species, but in the main comprehensive books on Palaearctic Chrysomelidae (Warchalowski 2010, Silfverberg 2010) *D.fukiensis* is still considered to be synonymous with *D.clavareaui*. These problems arose because the syntype series was neither accessible to Askevold nor to Cong and Yu. Today, the type series of *D.fukiensis* is stored at the Natural History Museum in Basel and it has been possible at last to examine it.

## Materials and methods

### Abbreviations of collections

**ASIZ** Academia Sinica, Institute of Zoology, Beijing, China

**CASC** California Academy of Science, San Francisco

**CMIC** Natural History Museum and Institute Chiba, Japan

**GBIF** Global Biodiversity Information Facility, https://www.gbif.org/

**IBNM** Ibaraki Nature Museum, Japan

**ISAC** coll. IS Askevold, Florida

**NHMB** Natural History Museum Basel, Switzerland

**NHMW** Natural History Museum Vienna, Austria

**NSMK** National Science Museum of Korea, Daejeon, South Korea

**MNHN** Muséum National d’Histoire Naturelle, Paris

**SDEI** Senckenberg German Entomological Institute, Müncheberg, Germany

**USNM** United States National Museum, Washington D.C., US

**ZSMC** Zoological State Collection, Munich, Germany

### Type specimens

#### Donacia clavareaui

Taxon classificationAnimaliaColeopteraChrysomelidae

Jacobson, 1906

##### Type locality.

Russia: Buryatia, Kjachta, 50°21'N, 106°27'E

##### Holotype.

MNHN EC2130: ♂ “Kjachta Siberie par Götzelmann [Clavareau’s handwriting]/ Donaciaclavareaui TYPE Jacob. [Clavareau’s handwriting]/TYPE [red, added by N Berti]/Museum Paris coll. H. Clavareau 1932/ Donaciaclavareaui Jac. ♂ typ. G. Jacobson det.”

##### Photograph of type specimen examined.

https://science.mnhn.fr/institution/mnhn/collection/ec/item/ec2130?listIndex=1&listCount=6 [26.11.2018]

#### Donacia fukiensis

Taxon classificationAnimaliaColeopteraChrysomelidae

Goecke, 1944

##### Type locality.

China: Fukien [Fujian], Kuatun [≈10 km NNE of Shaowu], 27°24'N, 117°24'E, 2300 m a.s.l.

##### Lectotype

**Figures 1–4.**
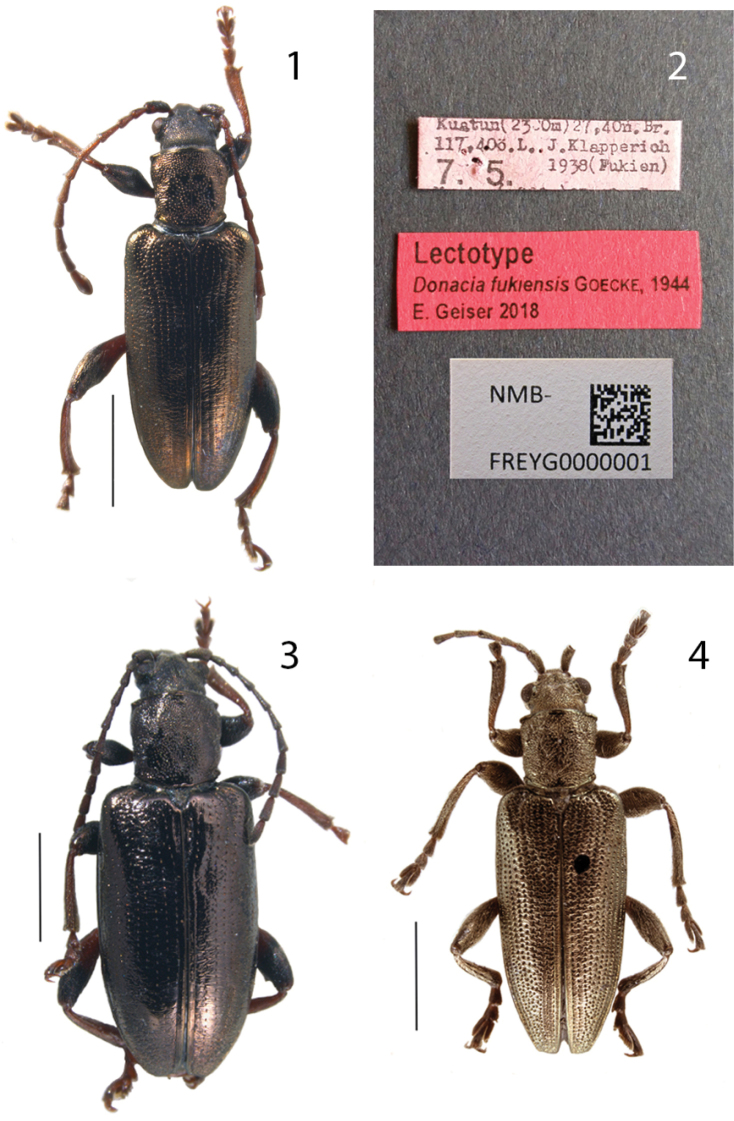
**1***Donaciafukiensis* Goecke, 1944, lectotype, male, China, Fujian, Kuatun (NHMB) **2***D.fukiensis*, labels of lectotype **3***D.fukiensis*, female, same data as lectotype **4***Donaciaclavareaui* Jacobson, 1906, male, China, Heilongjiang, Harbin (ZSMC). Scale bar 2 mm.

(here designated to fix the identity of the species). NMB-FREY0000001: ♂ “Kuatun (2300m) 27,40 n. Br. 117,40 ö. L.; J. Klapperich [leg.] 7.5.1938 (Fukien)”. NHMB in coll. Frey (Figs 1, 2)

##### Paralectotypes.

3 ♂, ♀♀ 7.5.1938, 3 ♂♂, ♀ 27.04.1938 (other data same as lectotype) (Fig. 3: ♀ from 7.5.1938 of this series)

Goecke did not designate a single type specimen; his description derives from seven syntypes, which are the specimens mentioned above. All of them are stored in the NHMB in coll. Frey.

#### Donacia kweilina

Taxon classificationAnimaliaColeopteraChrysomelidae

Chen, 1966

##### Type locality.

China: Guangxi, Kweilin, 25°16'55"N, 110°17'11"E.

##### Holotype.

♂, allotype: ♀, paratypes: 47 ♂♂, ♀♀ “Kwangsi: Kweilin (April-May, 1952)”

The type specimens are kept in ASIZ except for two paratypes in ISAC.

#### Donacia mediohirsuta

Taxon classificationAnimaliaColeopteraChrysomelidae

Chen, 1966

##### Type locality.

China: Yunnan, Shishong-Baana (Xishuangbanna), 22°1'N, 100°48'E, 1200 m a.s.l.

##### Holotype.

♀ “Yunnan: Shishong-Baana, 15.5.1958”

The type specimen is retained in ASIZ.

The characters of the type specimens of *D.kweilina* and *D.mediohirsuta* are analysed by the detailed first description of Chen (1966) and by further character descriptions mentioned in Cong and Yu (1997), who had examined these type specimens.

### Species record list

In Table 1 all records of these four *Donacia* species known to date are listed. The specimens indicated with “det. E. Geiser” or “vid. E. Geiser” were examined.

**Table 1.** List of specimen records of *Donaciaclavareaui*, *D.fukiensis*, *D.kweilina* and *D.mediohirsuta*

Species	Location	Lat.–Long.	Province	Country	Date	Qty	Legit	Determinavit	Coll.	Source
* D. clavareaui *	Kjachta	50°21.00'N; 106°27.00'E	Transbaikalia, Republic of Buryatia	Russia	–	1	Götzelmann	det. G. Jacobson 1906	MNHN	Photograph of type specimen (website MNHN)
	no details	–	Primorski krai (no more details)	Russia	–			det. Hayashi and Shiyake	–	Hayashi & Shiyake 2004, Bienkowski 2014
	a 30 km Suchebatora (= Süchbaatar)	50°14'N; 106°12'E	Selenge	Mongolia	–	1		det. L. Medvedev vid. E. Geiser 2018	ZSMC	specimen examined
	Mitanda, Katsuta	36°22'N; 140°33'E	Ibaraki-ken, Honshu	Japan	09.03.1988	10	Y. Narita	det. Y. Narita	IBNM	Narita 1991
	Urizura	36°30'N; 140°27'E	Ibaraki-ken, Honshu	Japan	09.01.1991	12	Y. Narita	det. Y. Narita	IBNM	Narita 2003
	Mito-shi	36°26.67'N; 140°26.18'E	Ibaraki-ken, Honshu	Japan	05.14.1986	8			IBNM	GBIF [22.10.2018]
	Iwasemachi, Nishiibaraki-gun	36°17'N; 140°25'E	Ibaraki-ken, Honshu	Japan	05.28.1995	2			IBNM	GBIF [22.10.2018]
	Ishioka-shi	36°13.13'N; 140°12.95'E	Ibaraki-ken, Honshu	Japan	07.05.1987	2			IBNM	GBIF [22.10.2018]
	Chiba	35°36'N; 140°6'E	Chibai-ken, Honshu	Japan	05.13.1988	4			CMIC	GBIF [22.10.2018]
	Chiba	35°36'N; 140°6'E	Chibai-ken, Honshu	Japan	06.10.1988	2			CMIC	GBIF [22.10.2018]
	Chiba	35°36'N; 140°6'E	Chibai-ken, Honshu	Japan	05.20.1987	4			CMIC	GBIF [22.10.2018]
	Harbin (Charbin)	45°45'N; 126°39'E	Heilongjiang (Amur-Province)	China	07.02.1950	1	W. Alin	det. H. Goecke 1952 vid. E. Geiser 2017	SDEI	Specimen examined
	Harbin (Charbin)	45°45'N; 126°39'E	Heilongjiang (Amur-Province)	China	26.–29.08.1953	2	Kardakoff	det. H. Goecke 1952 vid. E. Geiser 2018	NHMW	Specimen examined
	Harbin (Charbin)	45°45'N; 126°39'E	Heilongjiang (Amur-Province)	China	26.–29.08.1953	2	Kardakoff	det. A. Schneider 1956 vid. E. Geiser 2018	ZSMC	Specimen examined
	Harbin (Charbin)	45°45'N; 126°39'E	Heilongjiang (Amur-Province)	China	26.–29.08.1953	36	Kardakoff	det. H. Goecke 1956 vid. E. Geiser 2018	NHMB	Specimen examined
	Harbin (Charbin)	45°45'N; 126°39'E	Heilongjiang (Amur-Province)	China	06.06.1954	6		det. S. Cong & P. Yu	ASIZ	Cong and Yu 1997
* D. clavareaui *	Harbin (Charbin)	45°45'N; 126°39'E	Heilongjiang (Amur-Province)	China	06.06.1954	4		det. I. Askevold	ISAC	Cong and Yu 1997
	Imianpo, Harbin (Charbin)	45°45'N; 126°39'E	Heilongjiang (Amur-Province)	China	July 1938	1	Weymarn	det. S. Cong	CASC	Cong and Yu 1997
	Guanhsien	30°08'N; 102°56'E	Szechuan (2000 – 3000 ft)	China	1930	1	D.C. Graham	vid. Cong&Yu	USNM	Cong and Yu 1997
	Nulno-ri, Papyeong-myeon, Paju-shi	37°55.23'N; 126°51.96'E	Gyeonggi-do	South Korea	06.18.2015	6	S.L. An	det. S.L. An	NSMK	An 2018
* D. fukiensis *	Kuatun	27°24.00'N; 117°24.00'E	Fujian (2300 m a.s.l.)	China	04.07.1938	2	J. Klapperich	det. E. Geiser 2018	NHMB	Specimen examined
	Kuatun	27°24.00'N; 117°24.00'E	Fujian (2300 m a.s.l.)	China	04.12.1938	1	J. Klapperich	det. E. Geiser 2018	NHMB	Specimen examined
	Kuatun	27°24.00'N; 117°24.00'E	Fujian (2300 m a.s.l.)	China	04.25.1938	2	J. Klapperich	det. E. Geiser 2018	NHMB	Specimen examined
	Kuatun	27°24.00'N; 117°24.00'E	Fujian (2300 m a.s.l.)	China	04.27.1938	3	J. Klapperich	(det. Goecke 1944) det E. Geiser 2018	NHMB	Paralectotypes examined
	Kuatun	27°24.00'N; 117°24.00'E	Fujian (2300 m a.s.l.)	China	05.07.1938	4	J. Klapperich	(det. Goecke 1944) det E. Geiser 2018	NHMB	Lectotype and paralectotypes examined
	Kuatun	27°24.00'N; 117°24.00'E	Fujian (2300 m a.s.l.)	China	05.11.1938	1	J. Klapperich	det. E. Geiser 2018	NHMB	Specimen examined
	Kuatun	27°24.00'N; 117°24.00'E	Fujian (2300 m a.s.l.)	China	05.24.1938	1	J. Klapperich	det. E. Geiser 2018	NHMB	Specimen examined
	Kuatun	27°24.00'N; 117°24.00'E	Fujian (2300 m a.s.l.)	China	05.07.1938	1	J. Klapperich	det. Goecke 1952 vid. E. Geiser 2017	SDEI	Specimen examined
	Huangkeng, Jiangyang	27°20'N; 118°7'E	Fujian	China	28.03.1960, 05.–12.04.1960	7	F. Pu	det. P. Yu	ASIZ	Cong and Yu 1997
	Jiangyang	27°20'N; 118°7'E	Fujian	China	–	1	F. Pu	det. I. Askevold	ISAC	Cong and Yu 1997
* D. kweilina *	Kweilin	25°16.92'N; 110°17.18'E	Guangxi	China	April-May 1952	47	–	det. S. Cong&Yu	ASIZ	Cong and Yu 1997
	Kweilin	25°16.92'N; 110°17.18'E	Guangxi	China	April-May 1952	2	–	det. I. Askevold	ISAC	Chen 1966, Cong and Yu 1997
* D. mediohirsuta *	Shishong-Baana (Xishuangbanna)	22°1.88'N, 100°50.29'E	Yunnan (1200 m a.s.l.)	China	05.15.1958	1	–	det. S. Chen; vid. Cong&Yu	ASIZ	Chen 1966, Cong and Yu 1997

## Results

### Taxonomic history

Jacobson (1906) described the species *D.clavareaui* from Kjachta (Russia) in south-east Siberia. It could be easily distinguished from all other *Donacia* species known by its pubescent pronotum combined with glabrous elytra. In the subsequent decades several *Donacia* specimens from East Asia where identified as *Donaciaclavareaui*.

In the 1940s Goecke, a world-renowned Donaciinae specialist, examined specimens of *D.clavareaui* in the collection of the Museum Alexander Koenig in Bonn (Germany). He recognized that the specimens from Fujian (south-east China) were different in some characters which are typical for species limitation in *Donacia*. In 1944 Goecke published the description of the new species *D.fukiensis* which he split from *D.clavareaui*.

The description of Jacobson (1906) as well as the description of Goecke (1944) are both very detailed. However, Goecke did not describe which were the critical different characters for the distinction of *D.fukiensis* from *D.clavareaui.* He also published no identification key. Both descriptions match with both species (see Appendix 1, 2). This resulted in many misidentifications of East Asian specimens.

In 1961 Gressitt and Kimoto published their comprehensive volume “The Chrysomelidae of China and Korea”. Because there were so many Chinese specimens misidentified as *D.fukiensis* they assumed that *D.clavareaui* was restricted to Siberia. Therefore their identification key contains only *D.fukiensis*. The characters they mention in their key are applicable to both species. Their key became famous and widespread. Subsequently almost all specimens of *D.clavareaui* outside Siberia were identified as *D.fukiensis* from then on.

Chen (1966) recognized that within *D.fukiensis*, some specimens have different characters. He split two new species, *D.kweilina* and *D.mediohirsuta*, off from what was actually still a mixture of the two species *D.clavareaui* and *D.fukiensis*.

In the 1980s Askevold worked on his comprehensive revision of the genus *Donacia*. He investigated the type specimen of *D.clavareaui* which has been stored in the collection of the MNHN Paris. He also intended to investigate the type specimen of *D.fukiensis* stored in the collection Goecke which was then part of the private Coleopterea Museum Frey in Tutzing, Bavaria. Due to the special situation of the Museum Frey (see next chapter) no research on type or other specimens was possible at that time. Therefore Askevold studied series of *D.fukiensis* from Japan and China, which in fact were *D.clavareaui*. He concluded that there are no differences to the type specimen of *D.clavareaui* (he was right!) and therefore erroneously synonymized *D.fukiensis* with *D.clavareaui*. In 1990 Askevold published his comprehensive revision of the genus *Donacia* which has been widely used as a reference since.

In the 1990s Cong and Yu worked on a list of the Donaciinae of China. They recognized some differences in the specimens labelled *D.clavareaui* from Fujian as compared with specimens from other parts of China (as Goecke did more than 50 years before). Therefore they intended to study the type specimens of *D.fukiensis* from Goecke in Museum Frey. At that time, once again no loan of specimens was possible, but for a short period during the quarrels about the Frey collection it was stored at the ZSMC (see next chapter). Martin Baehr, the curator of Coleoptera section in Munich was in charge; Cong and Yu wrote to Baehr and asked him to check some critical characters at the syntype specimens of *D.fukiensis*, and Baehr confirmed these characters. Cong and Yu (1997) therefore removed *D.fukiensis* from synonymy and published the first identification key to distinguish *D.clavareaui* and *D.fukiensis*; they also included *D.kweilina* Chen, 1966 and *D.mediohirsuta* Chen, 1966. They also published accurate distribution data of these four species as far as they were substantiated.

The third volume of Water Beetles of China was published by Jäch and Ji in 2003 with Konstantinov as the author of the chapter about aquatic Chrysomelidae (Konstantinov 2003). He refers to all four species mentioned above, but he compiled their distribution data from sources where *D.clavareaui* and *D.fukiensis* were confused, and so they are not reliable.

In 2010 two very important comprehensive studies on Chrysomelidae were published: the Identification Key of Palaearctic Chrysomelidae (Warchalowski 2010) and the sixth volume of the Catalogue of Palaearctic Coleoptera which contained the Chrysomelidae in which Silfverberg was the author of the chapter on the Donaciinae (Silfverberg 2010). Both books are very useful and are the results of enormous workloads of the authors. Warchalowski is a specialist for Alticini (Galerucinae, Chrysomelidae) and Silfverberg is a specialist for Criocerinae und Galerucinae. Both wrote the Donaciinae chapter as no Donaciinae specialist was available and they both referred to the last comprehensive work on Donaciinae (Askevold 1990); therefore *D.fukiensis* is treated as a synonym to *D.clavareaui* in both volumes.

In 2015 a global checklist on Donaciinae was published (Geiser 2015), based on Silfverberg (2010) for the Palaearctic species and *D.fukiensis* is treated as a synonym to *D.clavareaui* there, also.

In 2017 I visited the collection of the SDEI in Müncheberg, Germany, which contains specimens of *D.clavareaui* and *D.fukiensis*, both identified by Goecke in 1952. I saw immediately what Goecke and Cong and Yu had seen before: that these two specimens differ in characters which are typical for separate species of Donaciinae. Fortunately the type specimens are accessible now in the NHMB and it was possible to check the characters of the seven syntypes and to finally designate a lectotype.

### The Museum Georg Frey and its unusual situation from 1976 to 1997

Georg Frey (1902–1976) was the owner of a clothes-producing company (“Lodenfrey”). He had an ardent interest in beetles, and attended and paid for field trips worldwide to collect beetles; he also bought collections from specialists. Near his house in Tutzing (south of Munich, Bavaria, Germany) he established a private museum and employed up to five scientists and assistants. When the Donaciinae specialist Hans Goecke died in 1963 Georg Frey bought his famous collection containing many type specimens (Anonymous 1963, Evers 1963).

In the decades after the WWII scientific institutions like natural history museums had insufficient and often only provisional storage facilities. At the Museum Frey the Goecke collection was well maintained as Frey employed the then-Chrysomelidae specialists, Jan Bechyne and Gerhard Scherer. When Georg Frey died in 1976, a quarrel began in the Frey family. The sons of Georg Frey intended to donate the whole collection to the ZSMC, because that had been the will of their father they argued; but the widow of Georg Frey began negotiations and finally sold the whole collection to the Natural History Museum of Basel, Switzerland. This started a conflict which involved the Frey family, the Munich State collection, several Switzerland institutions, and German Government institutions. The latter declared this beetle collection a national treasure which must not be transferred outside the borders of Germany. In 1992 the widow died and the collection was clandestinely transferred to the ZSMC before the Basel Museum received information on her death. The legal dispute continued and from 1995 onwards the collection was stored in boxes in Weil am Rhein, Germany, a city near Basel at the Swiss border (Furth 1996). In 1997 it was confirmed that the Museum Basel was the legitimate owner of this beetle collection and it was then transferred there (see further details from the Basel perspective in “Käfer für Basel” [https://kaeferfuerbasel.ch/die-sammlung-georg-frey/]). These incidents were the reason that between 1976 and 1998 it was impossible for long periods to borrow specimens and even to visit the collection to examine it in situ.

### Character analysis of *Donaciaclavareaui* and *Donaciafukiensis*

Jacobson (1906) described *D.clavareaui* in Latin and Goecke (1944) described *D.fukiensis* in German, both languages being widely used in science at the time. For traceability the original descriptions and their translations are shown in Appendix 1, 2.

The head, antennae, legs, and pronota are very similar, but their elytra are strikingly different. The main character differences are

– Shape of the contour of the elytra

– Punctures of the elytra

– Elytral epipleura

– Elytral apex

– Female: last sternite

– Male: aedeagus

All these character differences are typical for species in the genus *Donacia*. There are some well-established species in *Donacia* which differ in much more subtle characters. Therefore it was correct that Cong and Yu (1997) re-established *D.fukiensis* as a valid species. Now that the type series of Goecke is available to scientists, I was able to designate a lectotype from the seven syntypes on which the description of Goecke had been based (Fig. 1).

### Character analysis of *Donaciakweilina*

Chen (1966) described *D.kweilina* and *D.mediohirsuta* which he separated from the mixture of *D.fukiensis* and *D.clavareaui*. The common character of these four taxa is the pubescent pronotum combined with glabrous elytra. The first description is published in Chinese and in English. For practical considerations only the English text is shown in Appendix 3 (for *D.kweilina*) and Appendix 4 (for *D.mediohirsuta*). *Donaciakweilina* is known only from the type series (Cong and Yu 1997). No further records are known.

In Table 3 the characters of *D.kweilina* are listed according to the original description by Chen (1966) and provided by Cong and Yu (1997), who examined the type specimens. My comments result from the examination of specimens of *D.clavareaui*.

**Table 2.** Common and different characters of *Donaciaclavareaui* and *Donaciafukiensis.* Each character was based on specimens indicated in Table 1.

	* D. clavareaui *		* D. fukiensis *
General	Medium sized, pitchy brown, dark bronze, shiny, antennae and legs partially reddish, hind femora don’t reach the apex of the elytra, hind femora claviform with acute tooth, pronotal disc with very fine hairs, elytra glabrous		
**Body**			
Shape	Habitus like typical *Donacia* (Fig. 4)		Habitus resembles *Plateumaris* (Fig. 1)
Sex difference	Males in general more slender and shorter than the females		
Colour	Dark metallic-bronze, greenish-bronze, metallic-cupreous		Shiny bronze
Colour of antennae and legs	Antennae and legs partially yellow, reddish or brown, the extent of the colour is very variable within specimens		
Ventral	Ventral hairs as usual on *Donacia*, density variable, the colour of the hairs depends on the lighting		
Size	♂ 6.5- 8.0 mm (avg: 7.5), ♀ 8.0-9.0 mm (avg: 8.5)		
**Head**			
Antennae lenght	Filiform, slender, almost half as long as the length of the body, in some male specimens reaching farther than the middle of the elytra		
Antennomeres	A2+A3 ≈ A1 ≈ A4 ≈ A5; A2 < A3		
	The length relations of the single segments to each other are quite variable. The basal parts of the antennomeres are rufous or yellow, the apical parts are dark and sometimes metallic, the ratio between the two colour parts shows a great variation among the specimens		
Antennal tubercles	The antennal tubercles are flattened, with a narrow groove between them		
Head disc	Head disc straight at front with a deep middle groove		
Calli	Calli indistinct, some specimens without calli		
Frons and eyes	Eyes wide apart, the frons width is four times the measured value of the eye width, with no difference between male and female specimens		
**Pronotum**			
Surface	Pronotum pubescent, with very fine hairs, on some specimens very difficult to be seen		
Surface	Pronotum finely and densely punctured (Fig. 5)	Irregularly punctured, in between the punctures shiny. Often the punctures are more dense in the anterior and posterior part than in the middle part. Density of the punctures shows a great variation between individual specimens (Fig. 6, 7)	
Shape	Almost quadratic, in some male specimens slightly longer than wide, in some female specimens wider than long. Anterior margin slightly convex, anterior angles well developed, anterior tubercles rather flat, only slightly protruding		
Scutellum	Scutellum with thin and short hairs		
**Elytra**			
Shape	Typically *Donacia*-shaped		Rather *Plateumaris*-shaped
General features	Approx. twice as long as wide, in most male specimens slightly longer than double width (ratio 2.1), in most female specimens slightly shorter (ratio 1.9) glabrous and shiny		
Impressions	Slightly visible only on some specimens		
Punctures and intervals	Punctures strong and deep, intervals distincly wrinkled (Fig. 8) interval ≈ 1x – 3x puncture diameter		Punctures very delicate, not deep, intervals only slightly wrinkled, very smooth (Fig. 9) interval ≈ 4x – 7x puncture diameter
Epipleura	Elytral epipleura approx. as wide or wider than 10^th^ interval (Fig. 10) Epipleuron : Interval = 1 : (>) 1		Elytral epipleura narrower than 10^th^ interval (Fig. 11) Epipleuron : Interval = 1 : (1.5 – 2)
Apex	Elytral apex truncated, the external angle slightly rounded (Fig. 12)		Elytral apex indistinctly truncated, evenly and widely rounded with very smooth outer and inner angles (Fig. 13)
**Abdomen**			
Pygidium	Distinctly arcuately emarginate		Truncated and slightly recessed in the middle
Male last sternite	Apex rectangularly truncated and triangularly impressed		Slightly impressed at the apical ridge
Female last sternite	Basic contour distinctive triangular (Fig. 14)		Basic contour convex without a distinctive peak and broadly rounded (Fig. 15)
**Legs**			
General	Strong legs, all femora clavate, especially at the ♂, at the ♀ mostly more slender, hind femora short, even at the ♂ they don’t reach the apex of the elytra by far. Posterior femora with a prominent tooth, which is often broader at the ♂, at the ♀ more slender and more acute. Legs partly reddish, some specimens with completely red anterior tibia, some specimens with rather dark legs		
Anterior Tibia	Anterior tibia shows a protruding tooth towards outward at the insertion of the tarsomere. *D.fukiensis*: Fig. 18		
	*D.clavareaui*: Fig. 4 and https://science.mnhn.fr/institution/mnhn/collection/ec/item/ec2130?listIndex=1&listCount=6 [26.11.2018]		
	It is clearly visible on most specimens, but on some indistinctly		
Tarsomeres	The 1^st^ and 3^rd^ tarsomere have approx. the same length, the 2^nd^ one is by a third shorter		
**Aedeagus**			
Shape	Aedeagus very straight, outer contours in frontal view rather parallel. Median lobe distinctly protruding: Fig. 19, 20, 21		Aedeagus more curved, thickened, narrowed towards the apex. Median lobe slightly protruding: Fig. 22, 23, 24

**Figures 5–7.**
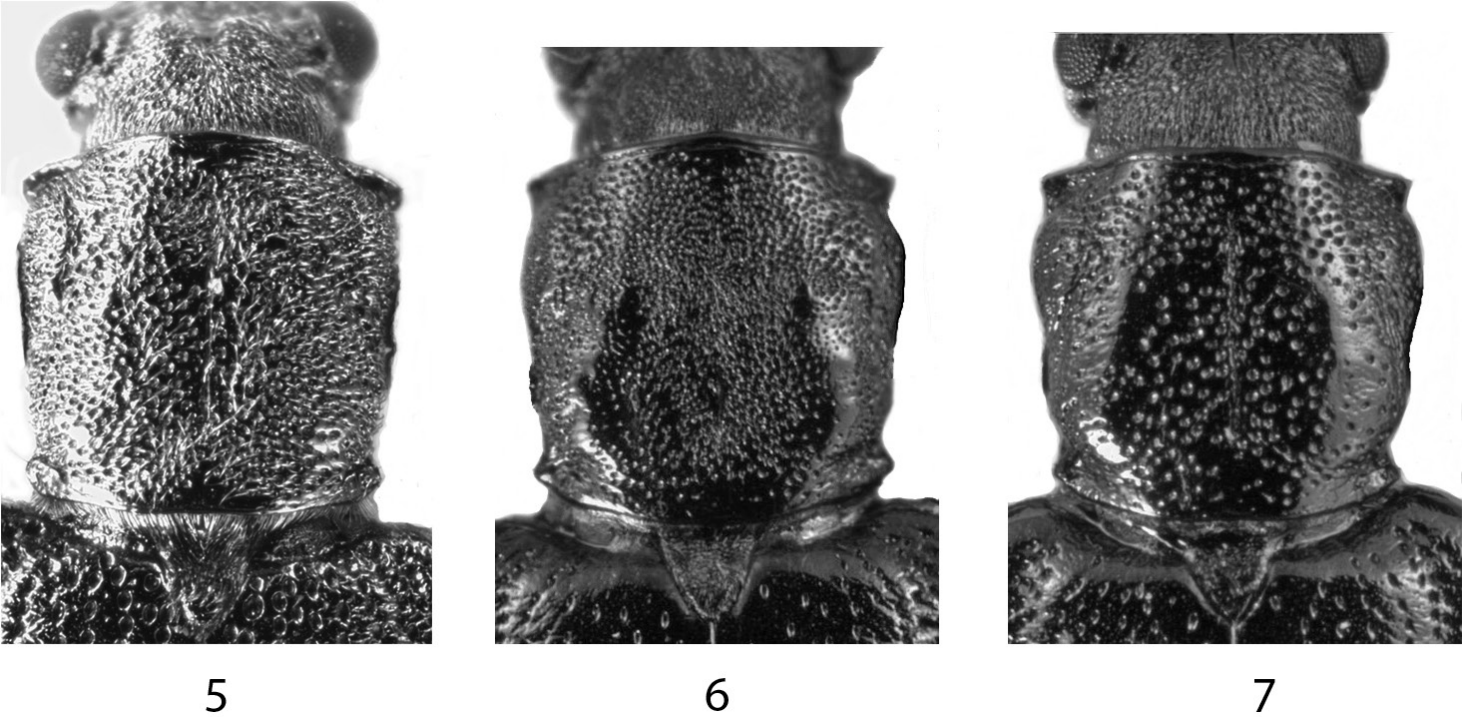
**5***Donaciaclavareaui*, Pronotum **6***D.fukiensis*, Pronotum densely punctured **7***D.fukiensis*, Pronotum irregularly punctured.

**Figures 8, 9.**
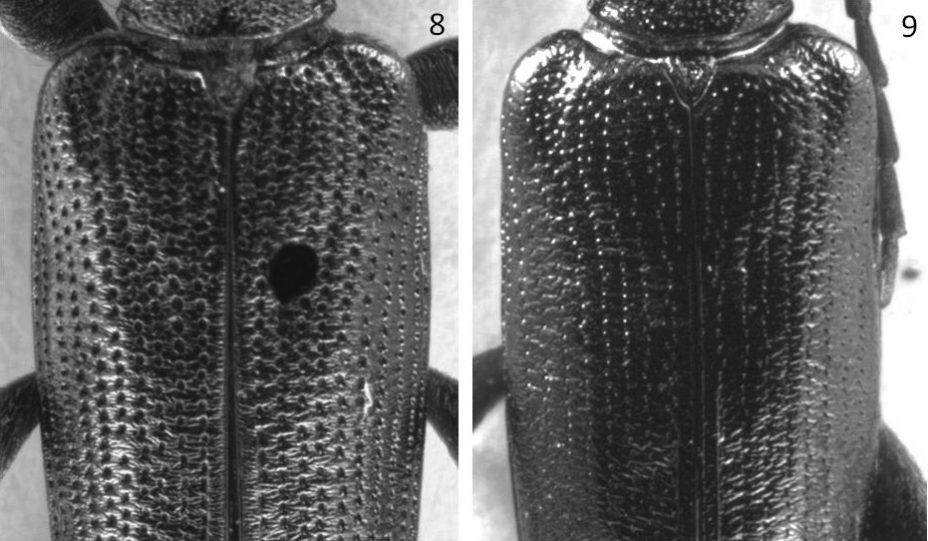
Elytral punctures. **8***Donaciaclavareaui***9***D.fukiensis*.

**Figures 10, 11.**
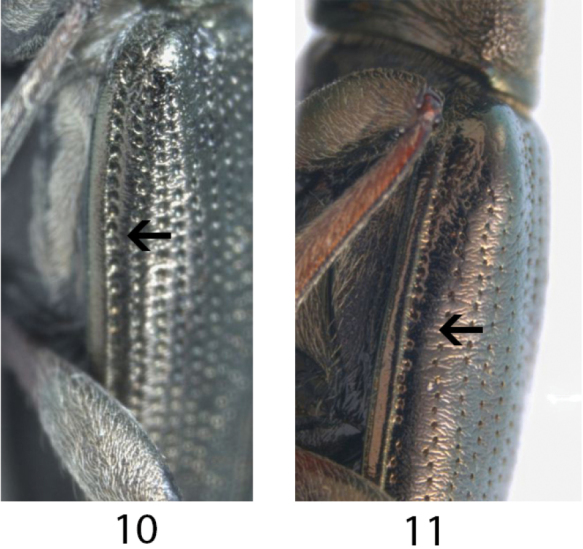
Elytral epipleuron. **10***Donaciaclavareaui*, 10^th^ interval narrower than epipleuron **11***D.fukiensis*, 10^th^ interval broader than epipleuron.

**Figures 12, 13.**
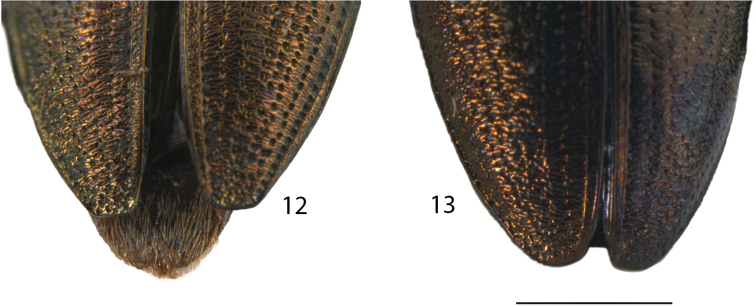
Elytral Apex. **12***Donaciaclavareaui***13***D.fukiensis.* Scale bar: 1 mm.

**Figures 14–17.**
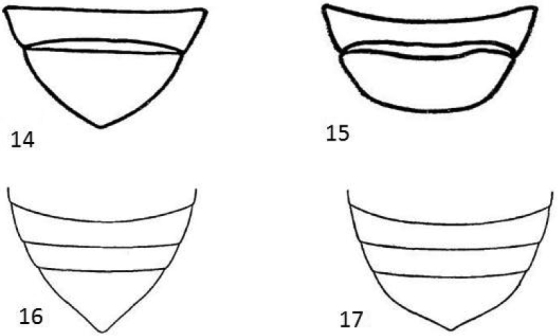
Female last sternite. **14***Donaciaclavareaui***15***D.fukiensis***16***Donaciakweilina***17***D.mediohirsuta* (Figs 14, 15 original drawings from Cong and Yu 1997, Figs 16, 17 original drawings from Chen 1966).

**Figures 18.**
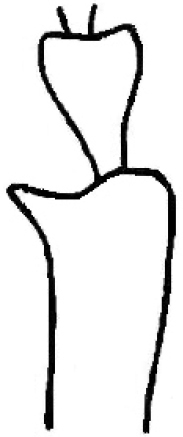
Anterior tibia: the protruding tooth towards outward at the insertion of the tarsomere is a common character of *Donaciaclavareaui* and *D.fukiensis* (original drawing from Goecke 1944).

**Figures 19–21.**
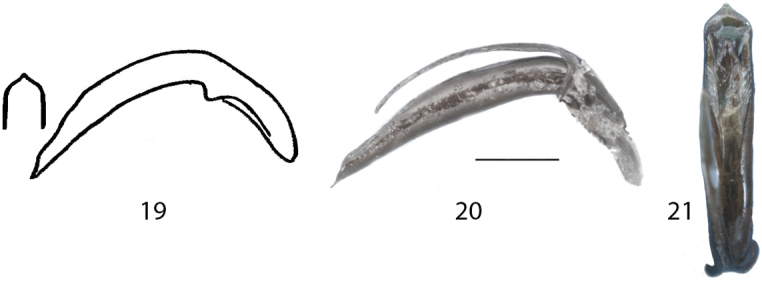
**19***Donaciaclavareaui* and *D.kweilina*, aedeagus (Original drawings from Cong and Yu 1997) **20***D.clavareaui*, aedeagus, lateral **21***D.clavareaui*, aedeagus, frontal. Scale bar: 0.5 mm.

**Figures 22–24.**
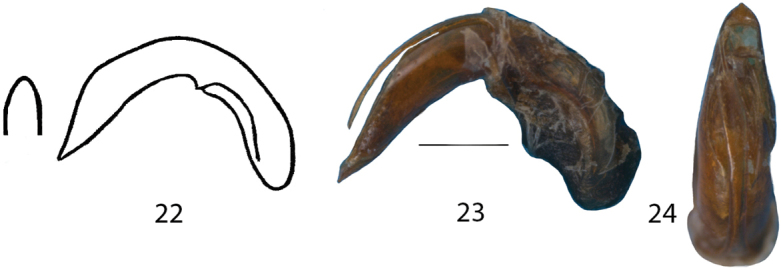
**22***Donaciafukiensis*, aedeagus (Original drawings from Cong and Yu 1997) **23***D.fukiensis*, aedeagus, lateral **24***D.fukiensis*, Aedeagus, frontal. Scale bar: 0.5 mm.

**Table 3.** Characters of *Donaciakweilina*.

Characters of *D.kweilina*	Comments
Colour aeneo-cupreous (♂, ♀) sometimes sky-blue (♂)	*D.clavareaui* is also aeneo-cupreous, sometimes blue males occur in Donaciinae species
Antennae and legs entirely deep coloured, not partly rufous	This occurs also in other *Donacia* species where most of the specimens have partially rufous antennae and legs; colour also very variable in *D.clavareaui*
Antennae: third segment slightly longer than second and distinctly shorter than fourth	same proportions of antennomeres in *D.clavareaui*
Head with four weak tubercles, the median longitudinal furrow deep and complete. Pronotum more thickly pubescent, very closely punctured, and covered with silvery hairs, the antero-lateral tubercles distinct, the angles fairly strongly produced. Elytra rather smooth on inner disc, the punctures oblong, the interstices broad, approx. 2–3 times as broad as the cross diameter of the punctures. Apex truncate with the outer angles broadly rounded.	All these characters can be clearly seen at the holotype specimen of *D.clavareaui*
Elytral epipleuron narrow and divided from outermost interval by sharp ridge throughout the entire length of elytra	This character is also clearly shown at *D.clavareaui* (Fig. 10)
Last abdominal segment of ♀ much longer and somewhat triangular in shape (Fig. 16)	Same typical shape as *D.clavareaui* (Fig. 14)
Hind femora (♂, ♀) broadly toothed beneath, the femora of ♂ not distinctly thicker than those of ♀	Same as *D.clavareaui*, thickness of hind femora variable
Aedeagus: Apex of median lobe cordiform (Cong and Yu 1997)	Cong and Yu (1997) refer to the same figure which shows the aedeagus of *D.clavareaui* (Fig. 19)
Length: 8 mm	Length of *D.clavareaui*: 6.5–9.0 mm

The characters which should distinguish *D.kweilina* from *D.clavareaui* are either the same or within the variations range of *D.clavareaui*. Therefore *D.kweilina* is a synonym of *D.clavareaui*.

### Character analysis of *Donaciamediohirsuta*

*Donaciamediohirsuta* is known only by the type specimen, a single female specimen from Yunnan, Shishong-Baana (Cong and Yu 1997). No further records are known. In Table 4 the characters of *D.mediohirsuta* are listed according to the original description by Chen (1966) and supplemented by Cong and Yu (1997), who have examined the type specimen.

**Table 4.** Characters of *Donaciamediohirsuta*.

**Characters of *D.mediohirsuta***	**Comments**
General colour cupreous	Same colour as *D.clavareaui*
Antennae with the terminal segments rufo-piceous, 3–5 segments partly rufous and partly piceous	Same as *D.clavareaui*
Third antennae segment distinctly longer than the second one, but slightly shorter than the fourth one	Same as *D.clavareaui*
Pronotum more transversal	In *D.clavareaui* the pronotum is as long as wide or slightly longer than wide; female specimens of *Donacia* sp. sometimes have a slightly broader pronotum
Pronotum finely pubescent only on the median groove	Pronotum pubescence varies in *D.clavareaui*
The longitudinal furrow of interocular area much deeper, extending uninterrupted to between the supra-antennal tubercles	These characters are distinctly visible at the holotype specimen of *D.clavareaui*
Anterior tibiae scarcely produced at apex	Variable; the protruded angle of the anterior tibia is mostly distinct, but in some specimens difficult to recognize
Hind femora (♀) very weekly toothed beneath	Variable in *Donacia* sp., especially female specimens have weak teeth in comparison with male specimens
Last abdominal sternite (♀) more strongly angulate at apex (Fig. 17)	Same typical shape as *D.clavareaui* (Fig. 14)
Length ♀: 8 mm	Length of *D.clavareaui* ♀: 8.0-9.0 mm

According to Cong and Yu (1997) this specimen resembles *D.kweilina* with only minor morphological differences. As shown in Table 4 the characters are identical or within the range of *D.clavareaui*. Therefore *D.mediohirsuta* is also a synonym of *D.clavareaui*.

### Identification key

**Table:** 

1	Pronotum with fine hairs on the disc (sometimes difficult to be seen, often more than 10 times magnification is necessary and lighting from different directions), elytra glabrous	**2**
–	Either pronotum and elytra are glabrous or both are pubescent	**other *Donacia* spp.**
2	Specimen from Nearctic region	***D.hirticollis* Kirby, 1837**
–	Specimen from Palaearctic region	**3**
3	Pronotum shape trapezoid, conical, anterior margin shorter than the posterior one, in male pronotum glabrous, here female only	***D.kraatzi* Weise, 1881**
–	Pronotum shape rectangular, anterior margin wider than or as wide as the posterior one	**4**
4	Pronotum as well as basal portion of elytra thickly covered with curved yellowish silver hairs, distal end of anterior tibia not produced laterally	***D.hirtihumeralis* Komiya & Kubota, 1987**
–	Pronotum covered with fine hairs, on elytra there are few hairs on the vertical surface anterior to humeral callus, distal end of anterior tibia produced laterally	**5**
5	Punctures on elytra rather strong, intervals one to two (sometimes three) times as wide as the diameter of the punctures, elytral epipleuron approx. as wide or wider than 10^th^ interval, elytral apex truncate (Fig. 12), the angles slightly rounded, female last sternite broadly triangular with posterior margin projected (Fig. 14), aedeagus rather straight and the median lobe cordiform with apex abruptly pointed (Figs 19, 20, 21)	***D.clavareaui* Jacobson, 1906**
–	Punctures on elytra rather fine, intervals three to seven times as wide as the diameter of the punctures, elytral epipleuron less wide (ca. ½ or ¾ of width) than 10^th^ interval, elytral apex rounded (Fig. 13), female last sternite broadly rounded (Fig. 15), aedeagus curved and the median lobe with slightly protruding apex (Figs 22, 23, 24)	***D.fukiensis* Goecke, 1944**

### Distribution

Due to the taxonomic problems there are only few reliable records, listed in Table 1.

**Figure 25.**
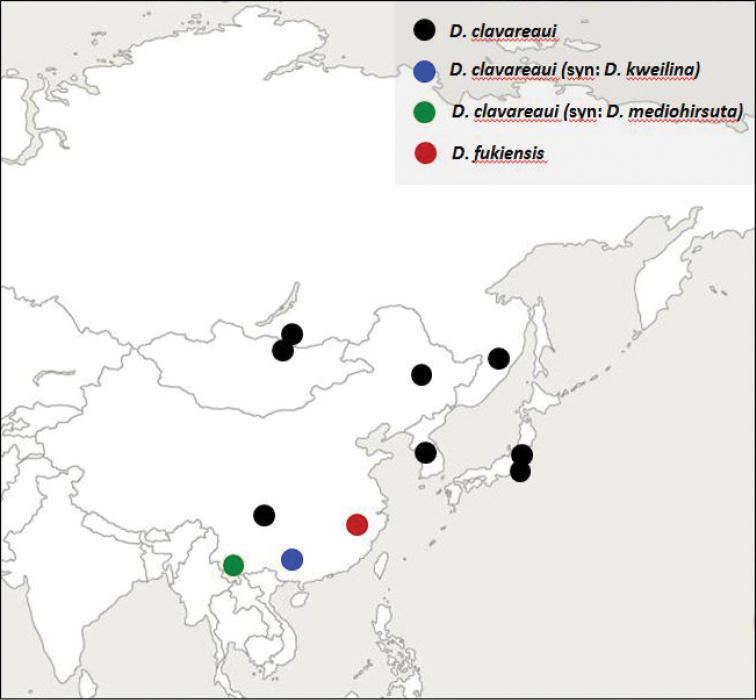
Distribution of records of the East Palaearctic species *Donaciaclavareaui* and *D.fukiensis*.

The known distribution of *D.clavareaui* is shown in Figure 25. Some dots represent more than one record and several nearby locations. The former *D.kweilina* and *D.mediohirsuta*, now synonymized with *D.clavareaui*, are shown by different coloured dots. The red dot represents the locations of *D.fukiensis*. No record of this species outside of Fujian is known. According to Fig. 25*D.clavareaui* occurs south of 50° latitude and east of 100° longitude. It is obvious that *D.clavareaui* must occur in many more locations than those shown in Fig. 25.

*Donacia* specimens are difficult to collect. The adults can be caught only during a period of a few weeks in late spring and early summer. This period shifts every year due to local weather conditions. Most rare species are found within groups of many specimens of other similar looking, more common *Donacia* species, and they are therefore often overlooked.

### Ecology

All Donaciinae species develop and feed on plants associated with water. As far as the food plants are known, *Donacia* species are monophagous or oligophagous. Some adults feed on pollen, mostly on Cyperaceae (Kleinschmidt and Kölsch 2011).The larvae live attached to the roots in the sediment. They breathe by piercing the aerenchyme of their food plant with two hollow abdominal stilettos, which are connected to their tracheal system.

The larva of *D.clavareaui* has been described by Narita (1991, 2003). The specimens were collected from roots of the Cyperaceae species *Scirpusfluviatilis* (Torr.) in Ibariki-ken in Honshu, Japan. According to Bienkowski (2014)*D.clavareaui* also feeds on *Isolepisfluitans* (L.) R.Br. (syn. *Scirpusfluitans*). An (2018) collected *D.clavareaui* in Korea on *Scirpusmaritimus* L. The food plants of *D.fukiensis*, *D.kweilina*, and *D.mediohirsuta* are unknown.

## Discussion

If specimens of *D.clavareaui* and *D.fukiensis* are compared directly, the differences are striking, especially of the elytra. Although the first descriptions of these species are comprehensive and detailed, they both described both species. Furthermore, it was not possible to create a reliable identification key without correctly identified specimens to hand. This created a vicious circle and caused decades of misidentifications, as well as the splitting of new species from a conglomerate of what was in fact two species. The situation was worsened by the inaccessibility of the type series of *D.fukiensis* in the Frey collection for a long period.

If specimens are identified incorrectly, all further studies on ecology and distribution are useless. In Figure 25 only reliable data of correctly identified specimens are used. In fact, it shows more the serendipity of the collectors than the reality of the distribution, but this is always the case within rare species. There are certainly more specimens stored in collections throughout the world, but they need to be examined and re-identified in light of the current classification as they may have been mistaken for other *Donacia* species. *Donaciafukiensis* may be also hidden within specimens of *Plateumaris*.

It is also very difficult to infer the distribution of *D.clavareaui* from its food plant. According to GBIF [https://www.gbif.org/species/2718286; 24.10.2018] *Scirpusfluviatilis* occurs outside of North America only in Japan and Korea and some spots on the east coast of Australia. The data provided by KewScience [https://wcsp.science.kew.org/namedetail.do?name_id=221898; 24.10.2018] indicate further records from New Zealand, but no records in Asia; GBIF shows only one record of *Isolepisfluitans* from Ceylon. *Scirpusmaritimus* is widespread, but there is only one record from China and none from Russia. It is very likely that *D.clavareaui* feeds on *Scirpus* sp. sensu lato.

Although both species are rare, I hope this paper will trigger some interest to examine the fauna more carefully during field trips in this area. If recent sample sites are known, it would be possible to find the food plant and larvae of *D.fukiensis* and to analyse the DNA of both species, to include them in the phylogenetic tree published by Kölsch and Pedersen (2008). Because the development of a pubescent upper side occurred several times in the evolution of the genus *Donacia* it is likely that they are not closely related.

## Supplementary Material

XML Treatment for Donacia clavareaui

XML Treatment for Donacia fukiensis

XML Treatment for Donacia kweilina

XML Treatment for Donacia mediohirsuta

## Appendix 1

**Table:** ***Donaciaclavareaui* Jacobson, 1906.** Original description in Latin and translation into English. The Latin text from Jacobson (1906) was translated in German by Remigius Geiser sen. The English translation results from this translated German text.

Character	Latin	English
General	Forma corporis coloreque superficiei supernae *D.bactriana* Weise turcestanicam et *D. Koenigi* m. caucasiam admonet, in systemate generis autem solum prope *D.intermediam* m. collocanda (**1**);	Owing to shape and surface colour as to be seen on the upper side it looks like *D.bactriana* Weise from Turkestan and like my *D. Koenigi* from the Caucasus, but in the system of the genus has to be placed near my *D.intermedia* only (**1**);
	(**1**) Cf. Opusculum meum in Ann. Mus. Zool. St.-Pétersb., V, 1899, pp. 4 et 7. Quam speciem novam prope *D.cineream* Herbst, *tomentosam* Ahr., *Kraatzi* Weise et *microcephalam* J. Daniel non pono, quod hae species pilositatem superficiei supernae talemcunque habent, ac pilositatem superficiei infernae.	(**1**) Compare my article in Ann. Mus. Zool. St.-Pétersburg, V, 1899, pp 4 and 7. This new species I don’t put near *D.cinerea* Herbst, *tomentosa* Ahr., *Kraatzi* Weise and *microcephala* J Daniel, because the surface of these species is as pubescent on the upper side as it is on the underside.
	nam ab omnibus speciebus, quae femora dentata habent, pronoto hirto tibiisque rufis unicoloribus facillime distinguenda;	because it can be easily distinguished from all other species with teeth on the femora by the pubescent pronotum and the uniformly coloured red tibiae;
	inter ceteras species pedibus antennisque rufovariegatis ornatas femoribus omnibus fortiter incrassatis posticisque dente sat valido atque acuto armatis agnoscitur. – ♂.	it is recognizable among the other species which are decorated with red patterned legs and antennae by the heavily thickened femora at each leg and the rather prominent and acute tooth on the hind femora. – ♂.
	Sat elongata, nitidula, subtus ut in *D.thalassina* Germ. dense flavaureo-pubescens [solum in prothoracis epipleuris pubescentia densa minus expansa, partem inferiorem occupante],	Quite longish, feebly shiny, underside with dense golden hairs as on *D.thalassina* Germ. [only at the epipleura of the prothorax the dense pubescence less spread and occupying the lower part],
General	aeneo-cuprea, antennarum *articulis omnibus* (apicalibus majore parte) basi, palpis omnino, mandibularum apice, labri margine apicali, trochanteribus, femorum triente basali ipsoque apice, tibiis omnibus tarsisque fere omnibus [superne nonnihil infuscatis] rufis.	metallic-cupreous, rufous are the basal parts of *all* antennae *segments* ([and] the major part of the apical ones), the whole palpae, the apical part of the mandibles, the apical margin of the labrum, the trochanters, the basal third and the end part of the femora, all tibiae and almost all [on the upper part slightly brownish] tarsomeres.
Head	Caput oculis sat magnis valdeque prominentibus;	The head with quite large and very protruding eyes;
	temporibus dense scopariis;	the tempora with dense, brush-like hairs;
	*canaliculo mediano profundo latoque*;	*the middle groove deep and broad*;
	*tuberculis frontalibus indistinctis*.	*the frontal calli indistinct.*
Antennae	Antennae dimidiam corporis longitudinem attingentes, tenues, articulo 2° tertio in ¼ breviore, art. 4° quinto vix perspicue breviore.	The antennae half as long as the length of the body, slender, the 2^nd^ segment by a quarter shorter than the third one, the 4^th^ one almost unrecognizably shorter than the fifth one.
Pronotum	Pronotum sericeum, latitudine aequilongum, postrorsum distincte subrectilineatim angustatum, medio nonnihil constrictum, callis lateralibus vix discretis, angulis anticis nonnihil incrassatis, sed extrorsum parum eminentibus;	Pronotum silky, as long as broad, towards the rear part distinctly almost rectangularly constricted, in the middle part slightly narrowed, lateral tubercles indistinct, anterior angles slightly thickened, but protruding only a little bit;
	canaliculo mediano haud profundo, solum medio distincto, antice posticeque omnino evanescente;	middle groove non deep, distinct only in the middle part, towards the front and backwards dissolving;
	disco nec profonde [sic!], nec fortiter punctato, *punctis omnibus piliferis*, medio majoribus sparsisque, antice posticeque minutis confertisque;	the disc punctured neither deeply nor strongly, *all punctures with hairs*, in the middle part larger and scattered, at the front and backwards small and dense;
	*pilis semierectis*, pallidis;	the hairs *half-erect*, pale;
	interspatiis puncturum [sic!] dense inaequaliterque rugulosis;	intervals between the punctures densely and irregularly wrinkled;
	rugulis irregularibus; proëpipleuris densissime irregulariter rugulosis ac punctulatis, subopacis, sparsim pilosulis.	wrinkles irregular; the pro-epipleura very densely irregularly wrinkled and finely punctured, almost matt, with scattered small hairs.
Scutellum	Scutellum dense ruguloso punctulatum atque tenuiter breviterque pubescens.	Scutellum dense wrinkly finely punctured and with thin and short hairs.
Elytra	Elytra quadrante basali subparallela, dein ad apicem gradatim rotundato-angustata, apice rectissime truncata, angulo exteriore parum rotundato;	The elytra in the basal quarter almost parallel, then toward the apex gradually roundly narrowed, the apex exactly rectangularly truncated, external angle slightly rounded;
	impressionibus, punctura et sculptura interspatiorum eadem ut in *bactriana*, solum interstitio primo postice rugulis transversis minus copiosis, minus expressis minusque regularibus.	impressions, puncture and texture of the intervals the same as with *bactriana*, only the first interval apically with fewer, lesser distinct and lesser regular transverse wrinkles.
Meta-sternum	Metasternum medio late excavatum (♂).	Metasternum with a broad hollow in the middle (♂).
Abdomen	Abdomen segmento primo medio longitudinaliter late impresso, segmento ultimo apice recte truncato et triangulariter impresso (♂).	The first segment of the abdomen in the middle longwise broadly impressed, the apex of the last segment rectangularly truncated and triangularly impressed (♂).
Pygidium	Pygidium distincte arcuato-emarginatum.	Pygidium distinctly arcuately emarginate.
Legs	Pedes fortes, femoribus omnibus incrassatis, posticis dente sat valido acutoque armatis deinque nonnihil crenulatis;	Strong legs, all femora thickened, the hind ones armed with a quite prominent and acute tooth and afterwards slightly notched;
	elytrorum apicem non attingentibus;	not reaching the apex of the elytra;
	tibiis posticis flexuosis, trientis primi apice vix inflato, absque crenulis.	hind tibiae curved, scarcely broadened at the end of the first third, without notches.
Size	Long. 8 mill.; lat. 2,6 mill. Habitat Provinciae Transbaicalicae urbem Kjachta in Sibira orientali (coll. Clavareau).	Length 8 mm; width 2.6 mm. Inhabits the town of Kjachta in the province of Transbaicalia in eastern Siberia (coll. Clavareau).

## Appendix 2

**Table:** ***Donaciafukiensis* Goecke, 1944.** Original description in German and translation into English.

Character	German	English
General	Mittelgroße einheitlich dunkelbronzefarbige glänzende Tiere mit äußerst fein behaartem Halsschild, die ♂♂ schlanker und kleiner als die ♀♀, deren Schenkel viel weniger keulig verdickt und deren 1. Hinterleibssegment nicht abgeplattet ist. Die Tiere sind im Habitus sehr einheitlich, in der Ausbildung der einzelnen Merkmale sehr variabel.	Medium sized uniform dark bronzy shiny animals with an extremelyfinely pubescent pronotum, the males more slender and shorter than the females, which have a much lesser clubbed thickened femur and their 1^st^ abdominal segment is not flattened. The animals’ habitus is very uniform, the formation of the single characters is very variable.
Head	**Oberkiefer** überragt die Oberlippe um etwas mehr als deren Länge, pechbraun, Kiefertaster gelb, bei einigen Stücken das letzte Glied an der Spitze braun.	**Mandibula** overlaps the labrum a bit more than its length, pitchy brown, maxillary palps yellow, at some specimens the last segment brown at the apex.
	**Oberlippe** etwa 2mal so breit wie lang, Vorderrand schwach konvex abgerundet, hinterer Rand mit langen Borsten, die bis über den Vorderrand ragen, Vorderrand mit kürzeren Borsten, dazwischen unbehaart.	**Labrum** ca. twice as broad as long, front margin slightly convexly rounded, basal margin with long setae, reaching beyond the front margin, front margin with shorter setae, in between without setae.
	**Kopfschild** vorn gerade, 2mal so breit wie die Seitenkante lang.	**Head plate** straight at front, twice as broad as the length of the side margin.
Anten-nae	Die **Fühler** sind fadenförmig, nicht sehr lang, ihr Ende überragt beim ♂ die Mitte der Flügeldecke, beim ♀ sind sie erheblich kürzer. 2. Glied am kürzesten, etwa halb so lang wie das 1., das 3. um 1/5 bis um die Hälfte länger als das 2., das 4. 1 ½ fach bis doppelt so lang als das zweite. Die einzelnen Glieder in ihrer Länge zueinander recht variabel. Fühlerglieder gelb bis dunkelbraun. 1. - 6. Glied mäßig dicht, 7. – 11. dichter behaart.	The **antennae** are filiform, not very long, in males reaching farer than the middle of the elytra, in females they are significantly shorter. 2^nd^ segment the shortest, about half as long as the 1^st^ one, the 3^rd^ one about one fifth to one half longer than the 2^nd^ one, the 4^th^ one is one and a half times to double the length of the second one. The length relations of the single segments are quite variable to each other. Antennomeres yellow to dark brown. The 1^st^ to 6^th^ one with moderately dense hairs, the 7^th^ to 11^th^ one with more densely packed hairs.
	Die **Fühlerhöcker** sind abgeplattet, dazwischen befindet sich eine schmale Furche, die Abplattung ist mehr oder weniger glänzend, fast ohne Punkte oder mäßig dicht punktiert, dahinter befindet sich eine mehr oder weniger deutliche Vertiefung, die gegen die Fühlerhöcker durch eine querliegende Kante abgesetzt ist. Die Stirnhöcker sind ziemlich flach und breit. Äußere Gruben flacher oder tiefer, innere Gruben schwach entwickelt.	The **antennal tubercles** are flattened, with a narrow groove between them, the flattened part is more or less shiny, almost without punctures or moderately densely punctured, behind it there is a more or less distinct depression, which is separated against the antennal tubercles by a transverse ridge. The calli are quite flattened and broad. Outer grooves more flattened or deeper, inner grooves shallow.
	**Stirn** mäßig dicht punktiert und behaart, glänzend.	**Frons** moderately densely punctured and pubescent, shiny.
	**Hals** hinter den Augen kaum verengt, **Schläfen** schwach entwickelt.	**Neck** scarcely narrowed behind the eyes, temples indistinct.
	**Augen** klein, weit auseinanderstehend.	**Eyes** small, wide apart.
Pro-notum	**Halsschild** an den vorderen Seitenhöckern am breitesten und etwa so breit wie in der Mitte lang. Bei einem Exemplar war das Halsschild allerdings erheblich länger.	Broadest part of the **pronotum** at the anterolateral tubercles and approximately as broad as its length in the midst. However the pronotum of one specimen was considerably longer.
	Die **Vorderecken** sind gut entwickelt, sie ragen aber weder über den Vorderrand noch über die Seitenhöcker vor.	The **anterior angles** are well developed, but neither protruding beyond the anterior margin nor the lateral tubercles.
	**Vorderrand** leicht konvex, gegen die Scheibe nicht, oder durch eine feine, oft unregelmäßige Linie abgesetzt.	**Anterior margin** slightly convex, not distinctly separated against the disc, or by a subtle, often irregular line.
	**Hinterecken** mehr oder weniger gut entwickelt, wenig vorragend.	**Posterior angles** more or less well developed, scarcely protruding.
	**Hinterrand** stark konvex, gegen die Scheibe nicht, oder durch eine feine, oft unregelmäßige Linie abgesetzt.	**Posterior margin** distinctly convex, not distinctly separated against the disc, or by a subtle, often irregular line.
	Die **Scheibe** des Halsschildes ist sehr variabel, gleichmäßig flach gewölbt, fast ohne Andeutung einer Mittelfurche oder auch abgeplattet und mit kräftiger Längsfurche. Die Mittelfurche erreicht weder den Vorderrand noch den Hinterrand, sie geht vorne oder hinten höchstens in eine sehr schwache oder nur angedeutete Vertiefung über.	The **disc** of the pronotum is very variable, evenly shallowly domed, almost without a hint of a central groove or flattened and with a distinct longitudinal groove. The central groove neither reaches the anterior nor the posterior margin, at the most it peters out to a shallow or only indistinct impression ahead or rearmost.
Pro-notum	Vordere **Seitenhöcker** deutlich, nach oben ein wenig oder kaum, gegen die Vorderecken kräftig, nach hinten schwach abgesetzt. Hintere Seitenhöcker schwach entwickelt. Wenig dicht, unregelmäßig punktiert, zwischen den Punkten glänzend. Oft ist die Punktierung vorne und hinten dichter als in der Mitte. Die Dichte der Punktierung ist aber bei den einzelnen Exemplaren sehr verschieden.	Anterior **lateral tubercles** distinct, against above slightly or scarcely, against the anterior angles distinctly, against backwards slightly separated. Posterior lateral tubercles poorly developed. Not densely, irregularly punctured, in between the punctures shiny. Often the punctures are more dense in the anterior and posterior part than in the middle part. But the density of the punctures is very different between single specimens.
	**Der Halsschild ist behaart.** Es befinden sich nämlich in den Punkten äußerst feine, kurze, sehr schwer sichtbare Borsten.	**The pronotum is pubescent**. For inside the punctures there are exceedingly delicate, short setae which are very difficult to be seen.
Prothorax	Die **Episternen** der Vorderbrust sind grob längs gerunzelt, der behaarte Fleck ist nur schwach behaart.	The **episterna** of prothorax are coarsely longitudinally wrinkled, the hairy patch is only feebly pubescent.
Elytra	**Flügeldecken** von vorn nach hinten schwach, zu den Seiten stärker gleichmäßig gewölbt, doppelt so lang wie zusammen breit. Die Seiten verlaufen parallel bis zum 2. Drittel und sind dann gleichmäßig zu den einzeln abgerundeten Enden gewölbt. Eine Abstutzung ist kaum angedeutet.	**Elytra** feebly domed from anterior to posterior, more distinctly and evenly towards the margins, twice as long as the breadth of both. Outer contour parallel from anterior to the second third, then evenly domed towards the singly rounded apices. Truncation indistinct.
	Die **Punktierung** ist sehr fein. Die Punkte sind länglich.	The **dotting** is very delicate. The punctures are longish.
	Die **Zwischenräume** sind flach und breit, glänzend mit flachen weit auseinander stehenden Querrunzeln und einer sehr feinen mehr oder weniger dichten Mikropunktur. Der 1. Zwischenraum ist fast glatt mit nur sehr schwacher Quer-, Längs- oder Schrägrunzelung und im hinteren Drittel auf beiden Seiten von einer linienförmigen Kante begrenzt.	The **intervals** are flattened and broad, shiny with flat, greatly separated transverse wrinkles and with very fine more or less dense micropuncture. The 1^st^ interval is almost glabrous with only weak transversal, longitudinal or diagonal wrinkles and margined on both sides with a ridge like a solid line in the last third.
	Die **Schulter** ist schwach entwickelt, ziemlich glänzend, schwach punktiert und gerunzelt.	The **humeral callus** is indistinct, rather lustrous, weakly punctured, and wrinkled.
	Der erste **Nahteindruck** ist bei einigen Stücken deutlich vorhanden, bei anderen kaum noch sichtbar. Andere Eindrücke außer der schwach entwickelten Schulterfurche fehlen.	The first **impression at the suture** is distinct only at some specimens, almost invisible at others. Other impressions are lacking besides the weakly developed humeral groove.
Meta-thorax	Die Unterseite der **Hinterbrust** ist beim ♂ herzförmig abgeplattet, beim ♀ gewölbt mit tiefer liegender Mittelfurche.	The underpart of the **metathorax** at the ♂ is heart-shaped and flattened, at the ♀ it is domed with a more prominent middle groove.
Abdominal segments	Das 1. **Hinterleibssegment** ist beim ♂ etwas, beim ♀ um die Hälfte länger als das 2. - 5. zusammen, es ist beim ♂ abgeplattet und etwas eingedrückt, beim ♀ gewölbt.	The 1^st^**abdominal segment** is slightly longer at the ♂, at the ♀ longer by the half than the 2^nd^ to 5^th^ together, at the ♂ flattened and slightly impressed, at the ♀ domed.
	Das letzte Segment ist beim ♂ an der Hinterkante leicht eingedrückt, beim ♀ konvex vorgezogen ohne eigentliche Spitze.	The last segment is slightly impressed at the apical ridge at the ♂, at the ♀ convexly protruding without a distinctive peak.
	Die Unterseite des Hinterleibes ist glänzend, mäßig dicht punktiert und behaart.	The underpart of the abdomen is shiny, moderately densely punctured and pubescent.
	Das **Pygidium** ist abgestutzt und in der Mitte schwach ausgebuchtet.	The **pygidium** is truncated and slightly recessed in the middle.
Legs	Die **Vorderschiene** ist an der Ansatzstelle der Tarse zahnförmig nach außen gebogen (siehe Abb. 6).	The **anterior tibia** shows a protruding tooth towards outward at the insertion of the tarsomere.
	Die **Hinterschenkel** sind kurz, sie erreichen auch beim ♂ das Flügeldeckenende bei weitem nicht, Vorder-, Mittel – und Hinterschenkel besonders beim ♂ stark keulig verdickt, beim ♀ schlanker.	The **posterior femora** are short, even at the ♂ they don’t reach the apex of the elytra by far, anterior, middle and posterior femora much thickened like clubs especially at the ♂, at the ♀ more slender.
	Hinterschenkel mit einem kräftigen Zahn, der beim ♂breiter, beim ♀ schmaler und spitzer ist (siehe Abb. 5).	Posterior femora with a prominent tooth, which is broader at the ♂, at the ♀ more slender and more acute.
	Das 1. und 3. Tarsenglied sind etwa gleich lang, das 2. um 1/3 kürzer.	The 1^st^ and 3^rd^ tarsomere have about the same length, the 2^nd^ one is by a third shorter.
Colour	Die Tiere sind einheitlich dunkel bronzefarben, nur die Fühler gelb bis dunkelbraun, die Schienen und Tarsen und die Hinterschenkel von der Basis bis zur Mittel hellbraun.	The animals are uniformly dark bronze, only the antennae yellow to dark brown, the tibae and tarsi and the hind femora light brown from the basal part to the middle.
Size	Länge: ♂ 7–8 mm, ♀ 9 mm. Breite: ♂ 2,4–2,6 mm, ♀ 3,5 mm.	Length: ♂ 7–8 mm, ♀ 9 mm. Width: ♂ 2.4–2.6 mm, ♀ 3.5 mm.
Locus typicus	Mir liegen vor 7 Exemplare aus dem Reichsmuseum **Alexander König** in Bonn, gesammelt am 27.4. und 7.5.1938 von Herrn **J. Klapperich** in Kuatun (Fukien, China) 27.40 nördl. Breite, 117.40 östl. Länge, in 2300 m Höhe.	There are 7 specimens on hand for me from the Reichsmuseum **Alexander König** in Bonn, collected at 27^th^ of April and 7^th^ of May 1938 by Mister **J. Klapperich** in Kuatun (Fukien, China) 27.40 northern latitude, 117.40 eastern longitude, at 2300 m a.s.l.

## Appendix 3

***Donaciakweilina* Chen, 1966**

Original description in English. The following species was described by Chen (1966) in Chinese and English. Only the English text and the illustration are provided here. The type specimens are stored in the collections of ASIZ.

“Closely related to *D.fukiensis* Goecke, distinguished by the pronotum much more thickly pubescent, the femora of ♂ not distinctly thicker than those of ♀ and the last abdominal segment of ♀ much longer and somewhat triangular in shape (Fig. 16).

Also allied to *D.clavareaui* Jacobson, but the antennae end legs entirely deep coloured, not partly rufous and the elytra rather more finely punctured with the interstices much broader and more sparingly and finely wrinkled.

Aeneo-cupreous (♂, ♀), sometimes sky-blue (♂). Antennae long and slender, metallic, the terminal segments black; third segment slightly longer than second and distinctly shorter than fourth. Head with four weak tubercles, the median longitudinal furrow deep and complete. Pronotum very closely punctured and covered with silvery hairs, the antero-lateral tubercles distinct, the angles fairly strongly produced. Elytra rather smooth on the inner disc, the punctures oblong, the interstices broad, about 2–3 times as broad as the cross diametre[sic!] of the punctures; apex truncate with the outer angles broadly rounded. Hind femora (♂, ♀) broadly toothed beneath.

Length: 6.8–8 mm.

Holotype ♂, allotype ♀, paratypes 47 ♂♂, ♀♀ Kwangsi: Kweilin (April-May, 1952).”

## Appendix 4

***Donaciamediohirsuta* Chen, 1966**

Original description in English. The following species was described by Chen (1966) in Chinese and English. Only the English text and the illustration are shown here. The type specimen is stored in the collections of ASIZ.

“Very like *D.fukiensis* Goecke, but with the pronotum more transversal, finely pubescent only on the median longitudinal area; the longitudinal furrow of interocular area much deeper, extending uninterrupted to between the supra-antennal tubercles; the anterior tibiae scarcely produced at apex; the hind femora (♀) very weakly toothed beneath and the last abdominal sternite (♀) more strongly angulate at apex (Fig. 17). General colour aeneo-cupreous. Antennae with the terminal segments rufo-piceous, 3–5 segments partly rufous and partly piceous, third segment distinctly longer than second, but slightly shorter than fourth.

Length: 8 mm

Holotype ♀ Yunnan: Shishong-Baana (1200 m, 15, May, 1958).”
